# A review of *Fasciolopsis buski* distribution and control in Indonesia

**DOI:** 10.14202/vetworld.2021.2757-2763

**Published:** 2021-10-26

**Authors:** Muhammad Rasyid Ridha, Liestiana Indriyati, Dicky Andiarsa, April Hari Wardhana

**Affiliations:** 1 Tanah Bumbu Unit for Health Research and Development, National Institute of Health Research and Development, Tanah Bumbu, South Kalimantan 72171, Indonesia; 2 Indonesian Research Center for Veterinary Science, Indonesian Agency for Agricultural Research and Development, Ministry of Agriculture Republic Indonesia, Bogor 16114, Indonesia; 3 Department of Parasitology, Faculty of Veterinary Medicine, Airlangga University, Surabaya, 60115, Indonesia.

**Keywords:** fasciolopsiasis, *Fasciolopsis buski*, Indonesia, trematode

## Abstract

Fasciolopsiasis is a parasitic infection caused by the flatworm *Fasciolopsis buski*. Since 1982, fasciolopsiasis has been reported in Indonesia’s Hulu Sungai Utara (HSU) Regency, South Kalimantan Province. Fasciolopsiasis occurs when contaminated raw or undercooked aquatic plants are consumed. Cercariae of the parasite encyst in a variety of aquatic plants and grow into metacercariae that infect and reproduce in the human intestine. Until now, treatment for *F. buski* infection in the HSU Regency has been comparatively short, with patients receiving only a single dose of praziquantel, 30 mg/kg body weight, without further observation. A long-term effort through health promotion activities and intensive health education, particularly for elementary school children enrolled in the School Health Program, is ongoing to help prevent fasciolopsiasis from spreading and to improve environmental sanitation. Through 2018, intervention efforts successfully reduced the incidence of *F. buski* infection. Sustaining surveillance and investigation of fasciolopsiasis, including identification of new cases and community education, is critical for the elimination of the parasite from Indonesia. This review describes the spread of *F. buski* and its possible impact on public health to understand the critical nature of continuing *F. buski* surveillance and control efforts.

## Introduction

Fasciolopsiasis is an intestinal disease caused by the zoonotic trematode, *Fasciolopsis buski*. The disease is considered neglected though infection with *F. buski* is endemic in several countries, including South and Central China [1], Taiwan [2], Southeast Asia (Thailand, Vietnam, Malaysia, Laos, and Kamboja) [3,4], Bangladesh [5], Japan [6], and Indonesia [7]. The parasite is estimated to infect approximately 10 million people worldwide [8].

**Figure-1:**
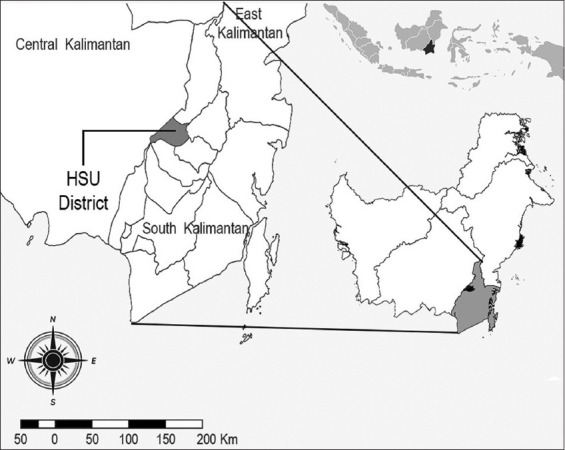
Endemic site of *Fasciolopsis buski* area in Indonesia [Source: Source: QGIS Nodebo 2.16.3].

The first case of fasciolopsiasis in Indonesia was reported in 1920, though the original case remains unidentified. Information on disease incidence is also limited. A remarkable report was published in 1982 for the Hulu Sungai Utara (HSU) Regency, South Kalimantan Province (Figure-1). Sungai Papuyu and Kalumpang Dalam villages in the Babirik subdistrict, Putat Atas and Padang Bangkal villages in the Sungai Pandan subdistrict, and Sarang Burung and Telaga Mas villages in the Danau Panggang subdistrict were identified as endemic areas for fasciolopsiasis. The incidence of infection was 27%, and 148 positive cases out of 548 people were examined. The highest incidence occurred among elementary school students, 79.1%, who presented with severe diarrhea [7]. The National Institute of Health Research and Development examined fasciolopsiasis occurrence in the villages of Sungai Papuyu, Kalumpang Dalam, Sarang Burung, Telaga Mas, Putat Atas, Padang Bangkal, and Sapala Bararawa and found 7.8% of 1555 residents carrying the parasite [9]. Residents in Kalumpang Dalam Village showed a notably high prevalence of infection (Table-1) [9-17]. This village has been a high prevalence area in the HSU for many years. HSU conducted the last survey in 2018; the survey was not repeated the following year due to the absence of cases. However, worm control was never resumed for animals that serve as reservoirs for the parasite. Thus, cases could still resurface. This article focuses on the spread of *F. buski* and its possible impact on public health in the HSU. Stakeholders can use this information to understand the critical nature of continuing *F. buski* surveillance and control efforts.

**Table-1:** *Fasciolopsiasis* survey in humans and snail in Indonesia between 1985 and 2018.

Years	No. of samples	Locations	Important findings (%)	Reference
Human survey				
1985/1986	548	HSU Regency	Positive 148 (27)	[9]
1986/1987	2.752	HSU Regency	Positive 504 (18.3)	[9]
1989/1990	2.451	HSU Regency	Positive 127 (5.1)	[9]
1990/1991	5.943	HSU Regency	Positive 266 (4.4)	[9]
1991/1992	3.279	HSU Regency	Positive 73 (2.2)	[9]
19992/1993	2.119	HSU Regency	Positive 94 (4.4)	[9]
1993/1994	2.204	HSU Regency	Positive 116 (5.2)	[9]
1994/1995	2.259	HSU Regency	Positive 167 (7.3)	[9]
1995/1996	2.286	HSU Regency	Positive 138 (6)	[9]
1996/1997	2.881	HSU Regency	Positive 105 (3.6)	[9]
1997/1998	2.580	HSU Regency	Positive 32 (1.2)	[9]
1998/1999	286	HSU Regency	Positive 17 (5.9)	[9]
1999/2000	3.838	HSU Regency	Positive 95 (2.4)	[9]
2000	2.724	HSU Regency	Positive 60 (2.2)	[9]
2001	614	HSU regency	Positive 14 (2.2)	[9]
2002/2003	274	Putat Atas Village, Sungai Pandan District, HSU Regency	Positive 16 (5.8)	[10]
2002/2003	190	Padang Bangkal Village, Sungai Pandan District, HSU Regency	Positive 18 (9.5)	[10]
2002/2003	223	Kalumpang Dalam Village, Babirik District, HSU Regency	Positive 34 (15.2)	[10]
2002/2003	236	Sungai Papuyu Village, Babirik District, HSU Regency	Positive 20 (8.5)	[10]
2002/2003	371	Telaga Mas Village, Danau Panggang District, HSU Regency	Positive 26 (7)	[10]
2002/2003	226	Sarang Burung Village, Danau Panggang District, HSU Regency	Positive 7 (3.1)	[10]
2002/2003	35	Sapala Bararawa Village, Danau Panggang District, HSU Regency	Positive (-)	[10]
2006	227	Kalumpang Dalam Village Babirik District, HSU Regency	Positive 11 (19.1)	[11]
2008/2009	161	Kalumpang Dalam Village, Babirik District, HSU Regency	Positive 3 (2)	[12]
2009	69	Sungai Papuyu Village, Babirik District, HSU Regency	Positive 7 (6.14)	[13]
2010	110	Babirik District, HSU Regency	Positive 5 (4.5)	[14]
2012	396	Sungai Papuyu and Kalumpang Dalam Villages, Babirik District, HSU Regency	Positive 11 (2.78)	[15]
2013	192	HSU Regency	Positive 1 (0.525)	[15]
2018	55	Sungai Papuyu Village, Babirik District, HSU Regency	Positive 1 (1,8)	[16]
Snail survey				
2012-2013	50 snails/ genus	Sungai Papuyu and Kalumpang Dalam Villages, Babirik District, HSU Regency	*Echinostome cercariae* were found in snail *Indoplanorbis* and *Echinostome cercariae*; *Strigea cercariae*; and *Obscuromicrocercous* *cercariae* were detected in snail *Lymnaea.*	[17]
2014	-	Sungai Papuyu and Kalumpang Dalam Villages, Babirik District, HSU Regency	*Echinostome cercariae* were found in snail *Lymnaea* and *Indoplanorbis* by PCR method	[17]

HSU=Hulu Sungai Utara, PCR=Polymerase chain reaction

This review updates *F. buski* distribution and control methods in Indonesia. We identified studies through an automatic database search of the National Library of Medicine’s PubMed database and a manual search using Google Scholar. In addition, data from the South Kalimantan Provincial Health Office and the Republic of Indonesia’s Ministry of Health were searched and collected for analysis.

### *F. buski* Life Cycle

**Figure-2:**
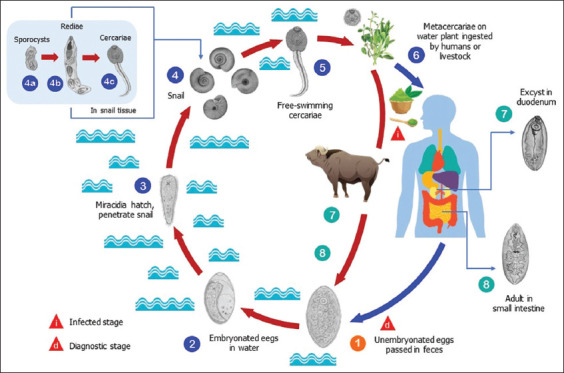
Life cycle of *Fasciolopsis buski* (modified CDC, pictures were extracted from Koan Nakagawa and freepik.com) [18,19].

*F. buski* is the cause of fasciolopsiasis. The parasite is the world’s largest trematode. Humans are its definitive host, and animals serve as reservoir hosts (HR). The worm lives and reproduces in the intestine and does not invade systemically. The life cycle of *F. buski* requires two intermediate hosts, HP I – a freshwater snail and HP II – aquatic plants (Figure-2) [18,19].

Fasciolopsiasis occurs in distinct reservoir hosts in different regions. Pigs are the most common reservoir in Kwangtung Province, China [20], and Southeast India [21]. Swamp buffalo, ducks, and chickens are also suspected reservoir hosts [22]. Swamp buffalo are kept by residents in several areas of endemic fasciolopsiasis in the HSU, most notably Sapala Bararawa and Sungai Pandan villages, raising concerns that they carry the parasite. In other areas, particularly in Kalumpang Dalam Village, Alabio ducks and chickens serve as HR.

Freshwater snails, *Lymnaea* spp., and *Indoplanorbis* spp., as HP I hosts, were confirmed by polymerase chain reaction [17]. *F. buski* eggs develop into miracidia that infect suitable freshwater snails and develop into sporocysts, redia, and cercariae. The latter escape from the snail and encyst in suitable aquatic plants [23]. Fasciolopsiasis occurs in humans when raw or undercooked aquatic vegetation is consumed.

Encysted cercariae develop into metacercariae, the infective form of the parasite for humans, in a variety of aquatic plants [24]. Several such plants that likely act as HP II hosts for fasciolopsiasis in the HSU are grown and commonly consumed by residents in areas where the parasite is endemic, especially lotus tubers and stems: Tatanding (*Nymphaea alba* and *Nymphaea lotus*), *susupan* (*Mimosa* spp.), *kangkung* (*Ipomea aquatica*), *genjer/patiul* (*Limnocharis flava*), *kalakai/pakis* (*Stenochlaena palustris*), *sulur*, and *tarati/palilak* (*Nymphaea*) [22].

## Distribution Update in Indonesia

*F. buski* surveillance activities began in 1985 in response to reports of children vomiting adult worms. The HSU Health Office conducted a massive surveillance program to ascertain infection prevalence and focus of intervention. Unfortunately, survey activities did not adequately define villages and district areas or attain program objectives [9]. The program did provide an overview of *Fasciolopsis* transmission in the HSU Regency from 1985 to 2001.

**Figure-3:**
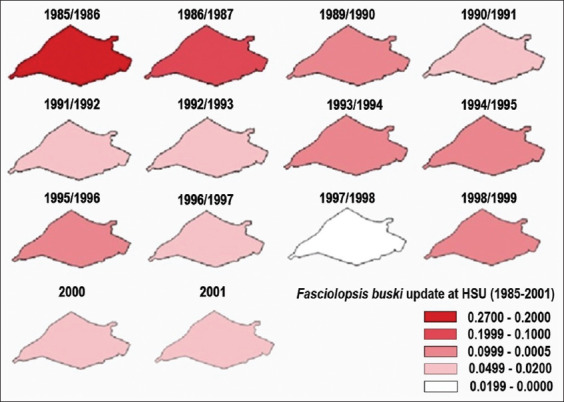
*Fasciolopsis buski* transmission updates 1985-2001 [Source: QGIS Nodebo 2.16.3].

In 1985 and 1986, fasciolopsiasis prevalence increased rapidly from 20% to 27% and then gradually declined to 1% in 1997 and 1998. Surprisingly, disease prevalence was higher in 1998 and 1999 and then hovered between 2% and 4% in 2001 (Figure-3).

**Figure-4:**
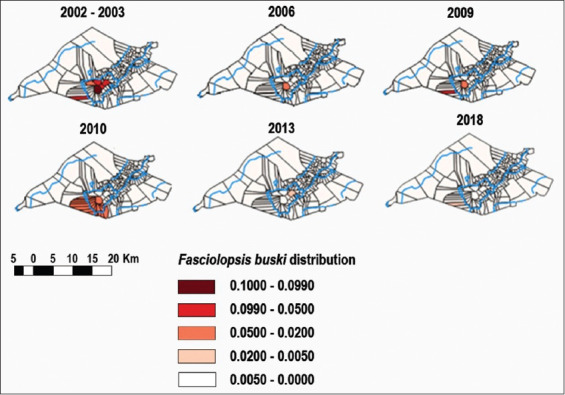
Distribution *Fasciolopsis buski* from 2002 to 2018 [Source: QGIS Nodebo 2.16.3].

In 2002, surveillance and research efforts shifted focus to areas suspected of being centers for parasite transmission. Transmission occurred exclusively within the Babirik and Danau Panggang subdistricts [10,17]. Both areas are bound by rivers and tributaries that flow into the Nagara. These conditions provide the parasite with an aquatic environment for free-living forms and a habitat where snails, as the HP I, thrive. In addition, these two areas are mostly swampy and inundated by rising river levels. The distribution of *F. buski* from 2002 to 2018 is depicted in Figure-4.

**Figure-5:**
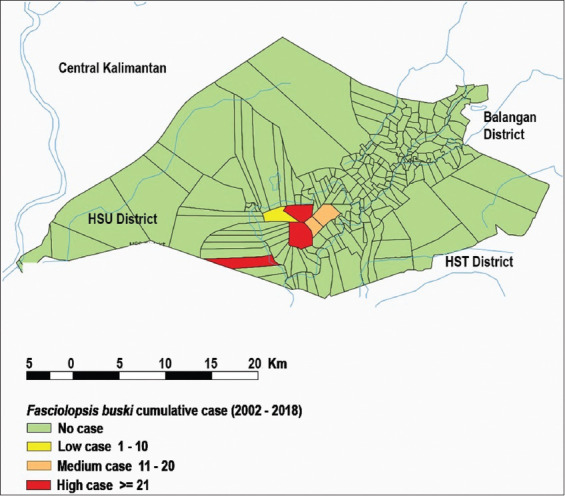
Cumulative cases of *Fasciolopsis buski* from 2002 to 2018 [Source: QGIS Nodebo 2.16.3].

From 2002 to 2018, fasciolopsiasis occurred locally in a few villages and three subdistricts in the HSU Regency (Figure-5). Babirik, Sungai Papuyu, and Telaga Mas villages exhibited the highest cumulative numbers of cases, Padang Bangkal and Putat Atas villages showed moderate numbers, and Sarang Burung village reported few cases.

## Control Methods

*F. buski* control in the HSU Regency involved treating patients with parasites with a single dose of praziquantel, 30 mg/kg. This effort was a short-term attempt to reduce the prevalence of *F. buski* in residents of the regency. Further, long-term efforts were made to improve environmental sanitation through health promotion campaigns and intensive health education, particularly for elementary school students [9]. One method for education is the distribution of comic books in the Banjar language that contains information about the parasite, its transmission, clinical disease symptoms, diagnosis, and consequences of infection. Importantly, the books also include information for treatment and prevention of the disease [25]. This effort increased children’s knowledge about *F. buski* infection by 14.8% and awareness of the importance of taking antiparasitic medication regularly by 11.2%. In addition, children reduced their consumption of aquatic plants. The community as a whole began consuming less aquatic vegetation, such as lotus tubers and *Caladium* spp. (30.0%), soup with raw fresh vegetables (8.8%), and lotus seeds, talipuk (35.0%) [25].

Further, a health promotion program with counseling was held in the HSU Regency using an Islamic religious lecture approach. This method was selected because Muslims constitute the majority of residents in *F. buski* endemic areas. The program was implemented in various Taklim groups at local mosques. Local religious leaders who had been trained by health officers delivered the lectures. This prevention approach was associated with the Islamic faith, as revealed in Al-Qur’an and Al Hadiths.

Many surveys were conducted to determine the effectiveness of promotional programs and to ascertain disease presence in the HSU Regency. Further, parasitological surveys were conducted to determine the dynamic prevalence of *F. buski* [9,26]. A separate investigation was focused on snails, reservoir hosts, and environmental assessments to identify factors that enhance infection and transmission of *F. buski* [17]. A survey and focus group discussions were used in some communities to assess the level of knowledge, attitude, and behavior [26].

## Discussion

Geographically, the HSU Regency is located at 2°27′0″S, 115°7′59.99″E. The regency is a lowland area with an elevation range of 0-7 m above sea level (masl) and a slope of 0-2%. Rainfall is influenced by climatic and geographical factors and rotation/confluence of air currents. The HSU receives a great deal of precipitation. Much of the area can be submerged throughout the year; more than 570 km^2^ of the total area of 892.7 km^2^ is swampy. The majority of the land is underutilized. Snails and aquatic vegetation thrive under these conditions.

Consumption of snails and raw aquatic plants, such as lotus shoots and tubers, water chestnuts, water caltrops, and lotus and water bamboo, consumption of untreated river or pond water, and frequent contact with livestock are all risk factors for *F. buski* infection [27,28]. An endemic district in the HSU Regency is associated with a community practice of consuming sweet lotus fruit and tubers (*Nymphaea* spp.), commonly referred to as “tanding” tubers. Exposure of children also occurs by ingesting raw water while swimming and playing after school [9,13]. The community consumes both lotus (*N. alba*) and bird lotus (*N. lotus*) [13]. Consumption of lotus tubers is a likely risk factor for *F. buski* infection in North India [21]. Water spinach (kangkung) is also a popular aquatic plant in the community and is linked with *F. buski* transmission in the HSU Regency [10] and Vietnam [29]. Consumption of metacercariae in aquatic plants was a primary factor in the outbreak in North India. However, consumption of snails also played a role [28]. Residents of the HSU Regency do not consume snails, but these mollusks are reservoir hosts that contribute significantly to the spread of the parasite. *Indoplanorbis* and *Lymnaea* spp. are likely cercariae carrier snail species [17].

Pigs and cows are reservoir hosts in the endemic area for *F. buski* in Bihar, India. Residents feed livestock lotus roots, which facilitates the spread of the parasite. In addition, high numbers of *F. buski* infections in Bihar were triggered by an appeal by a local nutritionist who suggested that snails could supply enough protein to support children’s growth [30]. If not cooked properly, the risk of infection from snails harboring *F. buski* is high.

Treatment of *F. buski* in the HSU Regency differs slightly from the treatment used in Bihar, India, most notably with respect to dose. In the HSU Regency, treatment consisted of a single dose of praziquantel 30 mg/kg body weight. In Bihar, India, this drug is administered 3 times daily at a dose of 25 mg/kg. The difference in dosage is most likely due to the region’s high endemicity and prevalence of *F. buski*. The parasite was found in 55 of 118 samples examined (parasite rate 46.6%). Adverse effects of praziquantel in Bihar included abdominal pain, nausea, and bloating followed by dizziness and headaches [30].

Due to limited data, surveillance efforts in the HSU district are necessary to obtain current data on prevalence and monitor *F. buski* elimination. Partnerships between the community and local governments must be strengthened to maximize prevention efforts.

## Conclusion

Fasciolopsiasis has been found in Indonesia since 1982 and only in the HSU Regency, Indonesia. The number of detected cases declined every year. Continuous monitoring efforts by local health authorities are still needed. Prevention and controlling of fasciolopsiasis will require a restriction in the consumption of high-risk foods and elimination of open defecation, especially in ponded water frequented by the public. Cross-sector partnerships could be strengthened with countermeasures against parasite infection.

## Authors’ Contributions

MRR: Conception and designed the study. LI: Collected the literature and organized the database. DA: Designed a spatial analysis. AHW: Adding concepts and literature studies. All of the authors wrote the manuscript and critically oversaw substantial revisions and approved the final manuscript.
